# Impact of an automated large vessel occlusion detection tool on clinical workflow and patient outcomes

**DOI:** 10.3389/fneur.2023.1179250

**Published:** 2023-05-25

**Authors:** Jennifer E. Soun, Anna Zolyan, Joel McLouth, Sebastian Elstrott, Masaki Nagamine, Conan Liang, Farideh H. Dehkordi-Vakil, Eleanor Chu, David Floriolli, Edward Kuoy, John Joseph, Nadine Abi-Jaoudeh, Peter D. Chang, Wengui Yu, Daniel S. Chow

**Affiliations:** ^1^Department of Radiological Sciences, University of California, Irvine, Orange, CA, United States; ^2^Department of Neurology, University of California, Irvine, Orange, CA, United States; ^3^Center for Statistical Consulting, School of Medicine, University of California, Irvine, Irvine, CA, United States; ^4^The Paul Merage School of Business, School of Medicine, University of California, Irvine, Irvine, CA, United States; ^5^Center for Artificial Intelligence in Diagnostic Medicine, University of California, Irvine, Irvine, CA, United States

**Keywords:** artificial intelligence, large vessel occlusion, stroke, machine learning, CT angiography

## Abstract

**Purpose:**

Automated large vessel occlusion (LVO) tools allow for prompt identification of positive LVO cases, but little is known about their role in acute stroke triage when implemented in a real-world setting. The purpose of this study was to evaluate the automated LVO detection tool’s impact on acute stroke workflow and clinical outcomes.

**Materials and methods:**

Consecutive patients with a computed tomography angiography (CTA) presenting with suspected acute ischemic stroke were compared before and after the implementation of an AI tool, RAPID LVO (RAPID 4.9, iSchemaView, Menlo Park, CA). Radiology CTA report turnaround times (TAT), door-to-treatment times, and the NIH stroke scale (NIHSS) after treatment were evaluated.

**Results:**

A total of 439 cases in the pre-AI group and 321 cases in the post-AI group were included, with 62 (14.12%) and 43 (13.40%) cases, respectively, receiving acute therapies. The AI tool demonstrated a sensitivity of 0.96, a specificity of 0.85, a negative predictive value of 0.99, and a positive predictive value of 0.53. Radiology CTA report TAT significantly improved post-AI (mean 30.58 min for pre-AI vs. 22 min for post-AI, *p* < 0.0005), notably at the resident level (*p* < 0.0003) but not at higher levels of expertise. There were no differences in door-to-treatment times, but the NIHSS at discharge was improved for the pre-AI group adjusted for confounders (parameter estimate = 3.97, *p* < 0.01).

**Conclusion:**

Implementation of an automated LVO detection tool improved radiology TAT but did not translate to improved stroke metrics and outcomes in a real-world setting.

## 1. Introduction

Stroke is one of the leading causes of death worldwide, and stroke morbidity is high with over half of the stroke victims left chronically disabled (1, 2). Acute stroke therapies including tissue plasminogen activator (tPA) and mechanical thrombectomy have been shown to improve clinical outcomes in several randomized clinical trials (3–11). The number needed to treat for revascularization to provide a clinical benefit in patients with large vessel occlusion (LVO) of the anterior circulation is less than three people (12). Given the impact of these life-saving measures, timely and accurate diagnosis of an LVO is critical to reducing patient morbidity and mortality.

Several commercially available automated tools for LVO detection on CT angiography (CTA) have been introduced into the clinical workspace (13, 14). The purpose of these tools is to triage positive LVO cases on a busy worklist through early identification and notification to the treatment team. These tools have demonstrated high accuracy, sensitivity, and specificity in retrospective studies (15–17).

However, the added value of these tools in real-world clinical settings is still unclear. Initial studies have shown that the implementation of an automated LVO detection tool is associated with reductions in transfer times, door-to-treatment times, and hospital and ICU stays, as well as improvements in clinical outcomes (18–20). Others have found low-to-moderate sensitivity and specificity and slower time to notification of these tools when implemented in the acute setting (21).

Moreover, little is known about the impact of an automated LVO detection tool on radiology workflow. Timely reporting of an LVO by a radiologist is essential for stroke triage, and the impact of integrating an automated LVO detection tool into stroke triage on CTA report turnaround times (TAT) still needs to be evaluated.

To address the gaps in knowledge regarding how these tools affect various components of acute stroke triage in the real-world setting, we evaluated the impact of an automated LVO detection tool, RAPID LVO (RAPID 4.9, iSchemaView, Menlo Park, CA), integrated into the workflow at a comprehensive stroke center. We hypothesized that the tool would (1) improve radiology CTA TAT and thereby (2) lead to improvements in stroke benchmarks and clinical outcomes.

## 2. Methods

### 2.1. Subjects

This retrospective study included consecutive patients presenting with suspected acute ischemic stroke who had a CTA at a comprehensive stroke center. The study period was between December 2019 and June 2020 for the pre-AI group and between December 2020 and June 2021 for the post-AI group. Inclusion criteria were as follows: (1) imaging performed within 24 h of symptom onset and (2) RAPID LVO output included with CTA acquisition (for the post-AI group). Exclusion criteria were as follows: (1) imaging acquired at outside facilities and (2) technically inadequate CTA (e.g., poor contrast bolus, significant motion, or other artifacts that would preclude evaluation by both human and automated assessment). Demographics and baseline stroke risk factors were recorded. The study was Health Insurance Portability and Accountability Act (HIPAA) compliant and was approved by the local institutional review board. A waiver of written consent was granted by the IRB.

### 2.2. Imaging and RAPID LVO acquisition

CTAs were acquired using three scanners from two vendors (Phillips and Siemens), including two 256-slice scanners with 128 detectors and a 128-slice scanner with 64 detectors. All CTA studies were performed as a single arteriovenous phase contrast study with a 60-mL intravenous contrast injection, an injection rate of 5 mL/s using bolus tracking triggered from the aortic arch, slice thickness 1 mm, and coverage of the aortic arch to the vertex.

**Figure 1:**
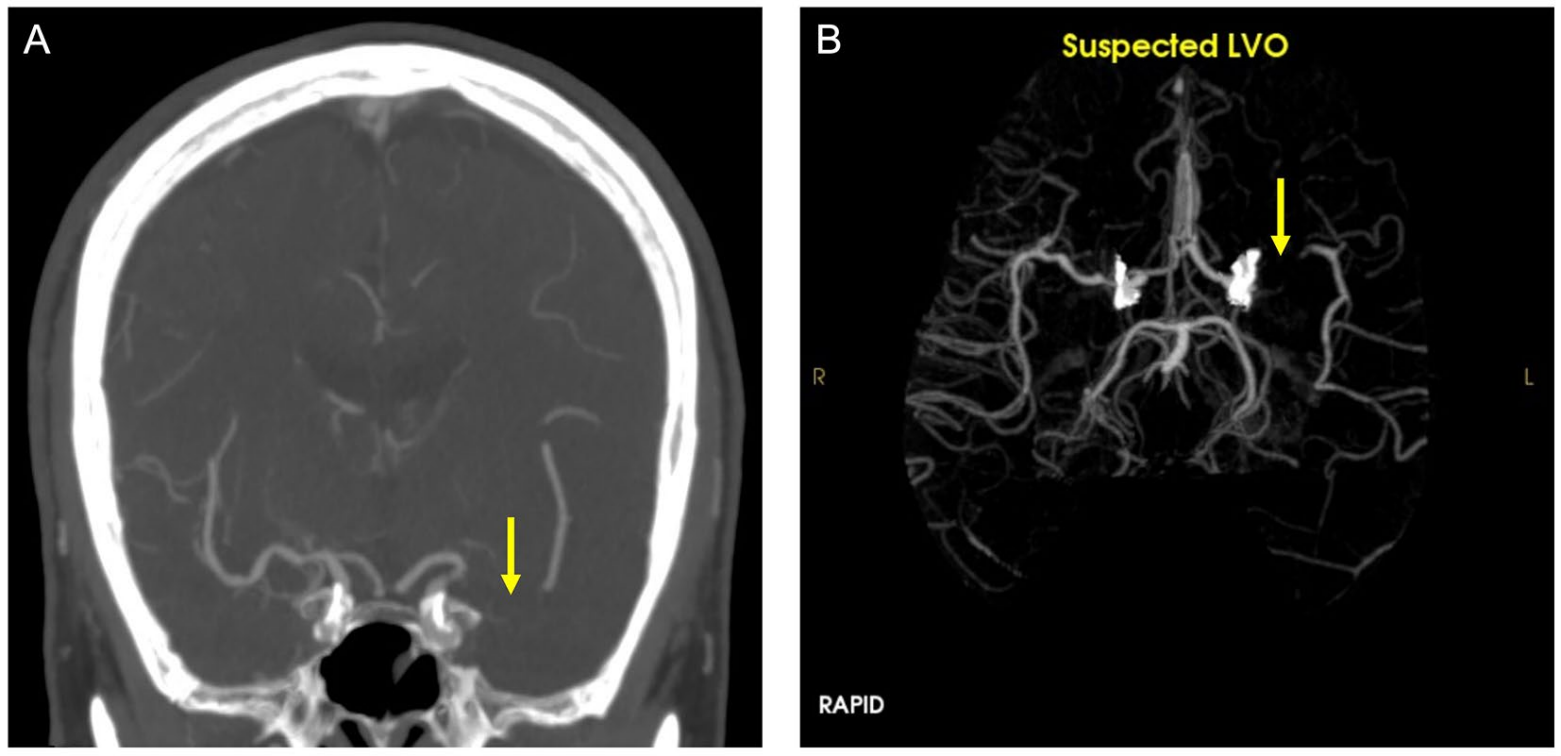
CTA **(A)** and RAPID LVO output **(B)** demonstrate a left middle cerebral artery occlusion (yellow arrow). This is a true positive confirmed by the CTA ground truth.

RAPID LVO is an FDA-approved automated LVO detection tool based on traditional machine learning techniques that can identify M1-MCA and intracranial ICA occlusions. The RAPID LVO algorithm which primarily relies on vessel density threshold assessment has been described previously (22). RAPID LVO was integrated into our hospital system in November 2020 and was available both in our Picture Archival and Communication System (PACS) and as a mobile or web application. All stroke team members, including the radiologist, stroke neurologist, and neuro-interventionalist, had access to RAPID LVO both in the PACS and on the mobile/web applications. RAPID LVO was implemented for a month prior to the initiation of this study to allow for adjustment to the software. An example of a screen capture of the RAPID LVO output accessible for viewing in PACS as well as mobile or web applications is shown in Figure 1.

### 2.3. Study design

#### 2.3.1. Rapid LVO performance

Assessment of LVO on CTA by radiologist’s reports served as the ground truth and was verified by a board-certified neuroradiologist (with 9 years of experience). In total, two additional neuroradiologists (with 9 and 11 years of experience) reviewed the complex cases where the ground truth was not clearly delineated. The presence and location of LVO were recorded. Performance metrics of RAPID LVO were analyzed for the post-AI group. Of note, RAPID LVO is not FDA-approved to evaluate the posterior circulation, anterior cerebral arteries, or M2/distal MCA occlusions. If an occlusion was not detected in these regions, it was considered a true negative case when evaluating RAPID LVO performance.

#### 2.3.2. Radiology CTA TAT

CTA report TAT was defined as the duration from study completion time (when the images are available to the radiologist) to the earlier time of either the report being available or read-back verification was provided for the clinicians. For radiology trainees, the preliminary report times served as the time the report was available to clinicians, and for attending radiologists, TAT was measured for completed report times. TAT was evaluated for all radiologists as well as by the level of expertise (subspecialty trained neuroradiologist, non-subspecialty trained radiologist, neuroradiology fellow, and radiology resident) and by LVO status. Negative TAT indicated that the report or read-back verification occurred earlier than the study completion time. TAT greater than 3 h was excluded from TAT analysis since these outliers were assumed to be due to systemic or technical errors.

#### 2.3.3. Stroke benchmarks and clinical outcomes

The AHA’s Get with the Guidelines*^®^*-Stroke reporting measures set benchmarks for important stroke metrics related to thrombectomy, including a 60 min door-to-needle, 90 min door-to-puncture, and 120 min door-to-revascularization times (23). These metrics were evaluated for both groups. We compared clinical outcomes including Thrombolysis in Cerebral Infarction (TICI) scores, NIH stroke scale (NIHSS) scores within 36 h post-therapy, NIHSS scores at discharge, and the difference in NIHSS scores between discharge and admission (ΔNIHSS). Mortality/significant morbidity [modified Rankin Scale (mRS) 5–6 at discharge] was also evaluated.

### 2.4. Statistical analyses

Statistical analyses were performed using the chi-squared test, student *t*-test, or Wilcoxon rank sum test when appropriate. For clinical outcomes, we used univariate analyses to determine which baseline characteristics and treatment variables were statistically significant and included the significant variables in multivariate regression analyses. Significance levels were set at a *p*-value of <0.05. Statistics were performed using SAS/STAT software.

## 3. Results

A total of 439 cases in the pre-AI group and 321 cases in the post-AI group met the inclusion criteria. There were 48 (10.93%) positive LVO cases in the pre-AI group and 47 (14.64%) positive LVO cases in the post-AI group. A total of 62 (14.12%) cases received acute therapies (tPA, thrombectomy, or both) in the pre-AI group and 43 (13.40%) cases in the post-AI group. For both groups, some negative LVO cases received tPA, and some positive cases were not treated based on standard treatment eligibility criteria. Baseline demographics, stroke risk factors, and lesion locations of patients determined by ground truth are shown in the Supplementary Table S1. The baseline characteristics were similar between both groups. For RAPID LVO performance metrics, accuracy was 0.87, sensitivity was 0.96, specificity was 0.85, positive predictive value was 0.53, and negative predictive value was 0.99.

Overall, CTA report TAT was significantly decreased in the post-AI group (mean ± SD, 30.58 min ± 29.85 for pre-AI and 22 min ± 35.07 for post-AI, *p* < 0.0005). When analyzed by the level of training, TAT was significantly decreased for resident interpreting cases using RAPID (31.70 min ± 29.63 pre-AI vs. 20.13 min ± 33.84 post-AI, *p* < 0.0003) but not significantly different for any higher level of expertise. TAT analyzed by LVO status (positive or negative) was significantly decreased in the post-AI group (32.67 ± 31.71 pre-AI vs. 12.13 ± 29.23 post-AI, *p* < 0.0007 for positive LVO cases and 30.24 ± 29.57 pre-AI vs. 23.74 ± 35.76 post-AI, *p* < 0.02 for negative LVO cases). TAT based on level of expertise and LVO status also decreased significantly for residents (31.48 ± 24.72 pre-AI vs. 7.14 ± 27.37 post-AI, *p* < 0.0006 for positive LVO cases and 31.74 ± 30.35 pre-AI vs. 22.26 ± 34.39 post-AI, *p* < 0.005 for negative LVO cases). Please see Table 1 for TAT details.

**Table 1:** Radiology report TAT for both groups.

	Pre-AI	Post-AI	*P* value
Overall (minutes), mean (SD)	30.58 (29.85)	22 (35.07)	0.0005*
Level of training
Resident	31.70 (29.63)	20.13 (33.84)	0.0003*
Neuroradiology fellow	37.25 (32.51)	32.44 (38.04)	0.70
Non-specialized attending	38.73 (37.62)	34.86 (23.17)	0.77
Neuroradiology attending	22.92 (27.62)	22.29 (36.59)	0.88
LVO status
Positive	32.67 (31.71)	12.13 (29.23)	0.0007*
Negative	30.24 (29.57)	23.74 (35.76)	0.02*
Level of training and LVO status
Resident
Positive	31.48 (24.72)	7.14 (27.37)	0.0006*
Negative	31.74 (30.35)	22.26 (34.39)	0.005*
Neuroradiology fellow
Positive	35.33 (41.08)	29.67 (48.79)	0.86
Negative	40.4 (28.95)	33.08 (37.55)	0.57
Non-specialized attending
Positive	n/a**	94 (7.07)	n/a
Negative	30.23 (32.55)	34.86 (23.17)	0.74
Neuroradiology attending
Positive	24.64 (42.97)	14.73 (28.61)	0.43
Negative	22.66 (24.99)	23.77 (37.89)	0.83

*Significance, *p* < 0.05; **Not applicable (no cases).

Stroke benchmarks, including door-to-needle, door-to-puncture, and door-to-revascularization times, and clinical outcomes, including NIHSS within 36 h post-therapy and at discharge, were not significantly different between both groups when compared using the Wilcoxon rank sum test (Table 2). ΔNIHSS was significantly different between the two groups, with the pre-AI group showing a greater NIHSS change compared to post-AI [median (IQR), 7 (2–13) pre-AI vs. 3 (0–7) post-AI, *p* < 0.03]. After adjusting for the effects of high cholesterol, heart disease, atrial fibrillation, therapies received, and NIHSS on admission, there was no significant difference in NIHSS within 36 h post-therapy based on multivariate analyses. For NIHSS at discharge adjusted for the same variables, there was a significant difference between the two groups (parameter estimate = 3.97, *p* < 0.01), with the post-AI group having higher NIHSS scores. Significant morbidity/mortality (mRS 5–6) was also not significantly different between the two groups.

**Table 2:** Stroke benchmarks and clinical outcomes of both groups.

	Pre-AI	Post-AI	*P* value
NIHSS on admission, median (IQR)	15 (9–23)	16 (7–22)	0.74
Treatment, no. (%)
tPA	15 (24.2)	9 (20.9)	0.71
Thrombectomy	34 (54.8)	27 (62.8)	
Both	13 (21)	7 (16.3)	
Door to image (minutes), median (IQR)	11 (8–20)	13 (7–20)	0.75
Door to intubation	64.5 (46–73)	69.5 (58–86)	0.15
Door to needle	37 (25.5–44)	42 (30–53)	0.38
Door to puncture	97 (80–107)	101 (90–113)	0.32
Door to revascularization	155 (123–197)	158 (131–191.5)	0.72
TICI, no. (%)			0.51
0	2 (4.3)	3 (8.8)	
1	1 (2.2)	0 (0)	
2A	4 (8.7)	4 (11.8)	
2B/C	18 (39.1)	17 (50)	
3	21 (45.7)	10 (29.4)	
NIHSS 36 h post-treatment	9.5 (5–18)	11 (2–20)	0.71
NIHSS at discharge	4.5 (1–11)	8 (1.5–20)	0.099
ΔNIHSS (discharge – admit)	7 (2–13)	3 (0–7)	0.03*
Significant morbidity/mortality (mRS 5–6), no. (%)	11 (17.74)	10 (23.26)	0.62

*Significance, *p* < 0.05.

## 4. Discussion

This study evaluated the impact of implementing an automated LVO detection tool on acute stroke triage. We found that RAPID LVO demonstrated high sensitivity (0.96), specificity (0.85), and negative predictive value (0.99) but a more modest positive predictive value (0.53). Overall CTA report TAT improved with RAPID LVO by almost 9 min (*p* < 0.0005). TAT improved with RAPID for residents (*p* < 0.0003) but not at higher levels of expertise. Subanalyses demonstrated reductions in report TAT of ~20 min for all positive LVO cases (*p* < 0.0007) and ~ 24 min for resident TAT of positive LVO cases (*p* < 0.0006). For stroke benchmarks, there were no significant differences in door-to-treatment times. Clinical outcomes were better in the pre-AI group for ΔNIHSS (*p* < 0.03) and NIHSS at discharge adjusted for potential confounders (*p* < 0.01).

**Figure 2:**
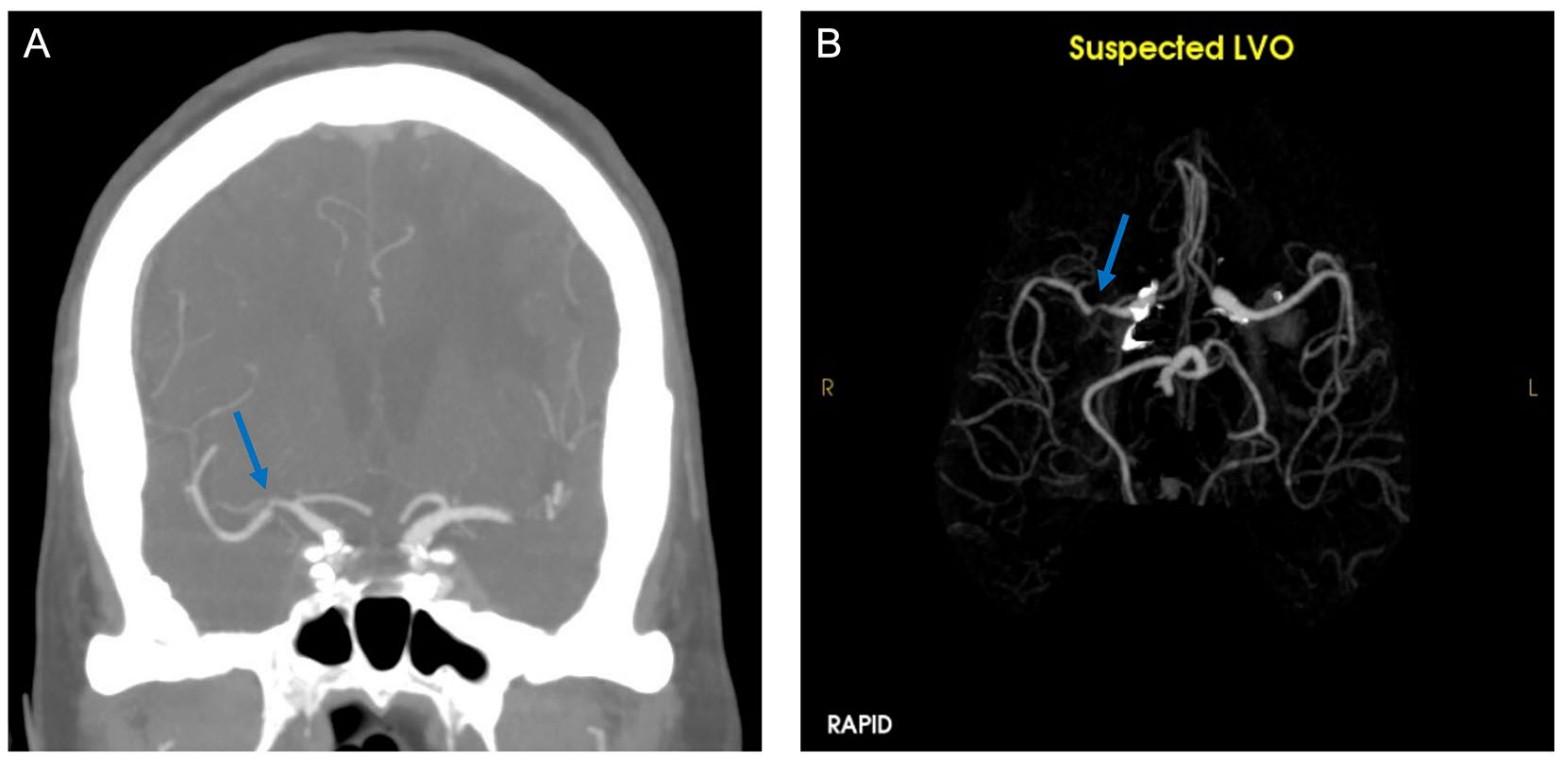
CTA **(A)** demonstrates stenosis of the right middle cerebral artery. RAPID LVO output **(B)** showed a suspected LVO (blue arrow).

Our performance metrics are similar to a prior study which demonstrated high sensitivity and lower specificity for RAPID LVO (17). The tool rarely misses an LVO which could be useful in the triage setting. However, the high false positives could lead to alert fatigue or unnecessary transfers (24, 25). In our study, false positives were most commonly seen in areas of caliber change (e.g., stenoses) (Figure 2). Over one-third of our patient cohort identified as Asian/Pacific Islander, and the prevalence of intracranial atherosclerosis is much higher in this population compared to other groups (26), possibly contributing to the high false positive rate that has not been seen in previous studies (20).

The implementation of RAPID LVO significantly improved CTA report TAT, notably at the resident level. This corroborates prior findings of automated tools improving report TAT for the detection of acute neuroimaging findings (27). The improvements in report TAT at the resident level may be due to increased reliance on the tool by less experienced readers. The significance of this result will be explored in future studies, as the impact of these tools on resident education is unknown.

There were no improvements in door-to-treatment times or immediate clinical outcomes for the post-AI group. This result differs from prior studies which showed improved stroke benchmarks using automated LVO detection tools (18–20). We also did not find any improvements in NIHSS or significant morbidity/mortality after therapies in the post-AI group, corroborating mixed outcomes from previous studies (18–20). This may be due to the nature of our stroke workflow, as the stroke team is on call 24/7 and accompanies the stroke patient to the CT scanner, often interpreting the CT/CTA at the scanner prior to receiving notification from the LVO detection tool. Of note, the post-AI group included patients during the highest COVID-19 hospitalizations which has been shown to impact stroke triage given the additional safety measures required and may have confounded door-to-treatment times although we found no significant differences in door-to-imaging times in 2021 (median 11 min) compared to 2020 (12 min) (28, 29).

Our mixed results highlight the limitations of an automated LVO detection tool at a comprehensive stroke center, namely that the stroke team members may not wait for the radiologist’s report for decision-making and that parallel processes for treatment decisions are occurring simultaneously based on the team’s clinical suspicion and own imaging interpretation. Additionally, the mobile notification of LVO status by RAPID LVO within a few minutes alerts the stroke team directly, potentially mitigating any link between CTA report TAT and outcomes. While previous studies have shown that the mobile application of an LVO detection tool was an independent predictor of reduced door-to-treatment times, the availability of the mobile application during this study did not support these findings (19).

While our study shows the diagnostic success of these tools in LVO screening and a reduction in CTA report TAT (hypothesis 1), it did not demonstrate clinical value beyond these metrics (hypothesis 2). Previous studies have shown the importance of negative studies in optimizing quality standards and addressing technical limitations (30, 31). Our study found that improved TAT alone does not translate to improved door-to-treatment times or patient outcomes. Although a partially negative result, this study raises important questions and demonstrates the need for further investigation about the optimal way to integrate these tools into radiology practice beyond detection. For example, the implementation of an LVO detection tool may be more valuable in a resource-or expertise-limited setting to rapidly triage positive cases for transfer and aid less experienced readers. Prior studies support this notion with a significant reduction in transfer times and door-to-treatment times in institutions with hub-and-spoke systems in place (18, 19).

Understanding the value of an automated LVO detection tool is increasingly relevant not only for patient care but also for financial incentives, as the Centers for Medicare and Medicaid Services recently granted a New Technology Add-on Payment (NTAP) for AI-based LVO detection software, which represents for the first time this reimbursement has been designated for an AI platform (32). This new reimbursement has accelerated the adoption of these tools into clinical practice (33). Ultimately, there remains a need to continue to ascertain the health benefits, not just technical success, of these tools to define care standards and mitigate inappropriate use.

There were limitations in our analysis, notably a retrospective analysis with a small sample size at a single center. Interpretation of positive and negative predictive values may be limited in this cohort, affected by factors such as single-center bias, selection bias, confounders, sample size limitations, temporal changes in stroke practices, and misclassification biases. Further evaluation of how the tool is used by various members of the stroke team is warranted. CT perfusion (CTP) was used for a small subset of patients with a delayed or unknown presentation to help discern treatment eligibility. In the future, this subset could be analyzed separately to determine if CTP confounded outcomes. Additionally, the heterogeneity of impact on outcomes from prior and current studies could be attributed to differences in study design, patient populations, and stroke triage practices. These differences highlight the possibility that an automated LVO detection tool may demonstrate utility in certain clinical environments but may have limitations if applied with a “one-size-fits-all” approach.

## 5. Conclusion

This study shows the potential utility of an automated LVO detection tool for stroke triage. The highly sensitive tool has utility in triaging patients who may require acute therapies by allowing for faster radiology turnaround times, but challenges remain, particularly in understanding how to translate the performance of the tool into meaningful clinical improvements.

## Data availability statement

The raw data supporting the conclusions of this article will be made available by the authors, without undue reservation.

## Ethics statement

The studies involving human participants were reviewed and approved by the University of California, Irvine Institutional Review Board. Written informed consent for participation was not required for this study in accordance with the national legislation and the institutional requirements.

## Author contributions

JS: study design, manuscript preparation, data gathering, and analysis. AZ, JM, SE, MN, and CL: data gathering and analysis. FD-V and PC: data analysis. EC, DF, and EK: data gathering. JJ and NA-J: study design. WY: study design and manuscript revision. DC: study design and manuscript revision. All authors contributed to the article and approved the submitted version.

## Funding

JS received funding from the Radiological Society of North America Research Scholar Grant.

## Conflict of interest

JS current research funding from Canon Medical. PC co-founder of and owns stock in Avicenna.AI, received past and current research funding from and is a paid consultant for Canon Medical, has a current grant from Novocure, has past research funding from GE, and is a paid consultant and speaker for Siemens. DC owns stock in Avicenna.ai and is a paid consultant for Canon Medical.

The remaining authors declare that the research was conducted in the absence of any commercial or financial relationships that could be construed as a potential conflict of interest.

## Publisher’s note

All claims expressed in this article are solely those of the authors and do not necessarily represent those of their affiliated organizations, or those of the publisher, the editors and the reviewers. Any product that may be evaluated in this article, or claim that may be made by its manufacturer, is not guaranteed or endorsed by the publisher.
