# Supplementary material for: Impact of an automated large vessel occlusion detection tool on clinical workflow and patient outcomes
Source: Front Neurol. 2023 May 25;14:1179250. doi: 10.3389/fneur.2023.1179250 (PMC10248058; doi:10.3389/fneur.2023.1179250)
Supplement: Supplementary file 1 [file Table_1.docx]

**Table S.1**: Baseline patient demographics and clinical characteristics of both groups.

| **Baseline characteristics** | **Pre-AI** | **Post-AI** | **P value** |
| --- | --- | --- | --- |
| Age, median (IQR), y | 71.5 (65-79) | 75 (65-83) | 0.31 |
| Female, no. (%) | 28 (45.2) | 22 (55.2) | 0.54 |
| Race, no. (%) |  |  |  |
| African American | 2 (3.2) | 1 (2.3) | 0.27 |
| Asian/Pacific Islander | 24(38.7) | 14(32.6) |  |
| Hispanic | 11(17.7) | 13(30.2) |  |
| White | 25(40.3) | 13(30.2) |  |
| Other/Did not specify | 0(0.0) | 2(4.7) |  |
| Atrial fibrillation, no. (%) | 30(48.4) | 17(39.5) | 0.37 |
| Diabetes, no. (%) | 25(40.3) | 15(34.9) | 0.57 |
| Heart Disease, no. (%) | 22(35.5) | 18(41.9) | 0.51 |
| High Cholesterol, no. (%) | 32(51.6) | 26(60.5) | 0.37 |
| Hypertension, no. (%) | 54(87.1) | 36(83.7) | 0.63 |
| Obesity, no. (%) | 6(9.7) | 10(23.3) | 0.06 |
| Prior Stroke, no. (%) | 15(24.2) | 10(23.3) | 0.9 |
| LVO locations, no. |  |  |  |
| ICA | 4 | 8 |  |
| M1 | 17 | 23 |  |
| M2 | 15 | 13 |  |
| ≥2 Lesions | 7 | 6 |  |
| A1 or posterior circulation | 5 | 5 |  |
